# The Use of Micro Retractor in Endoscopic Endonasal Posterior Pseudocapsule Resection of Pituitary Macroadenoma. Technical Note

**DOI:** 10.3389/fonc.2021.714342

**Published:** 2021-08-13

**Authors:** Tao Xie, Xiaobiao Zhang, Chenghui Qu, Chen Li

**Affiliations:** ^1^Department of Neurosurgery, Zhongshan Hospital, Fudan University, Shanghai, China; ^2^Digital Medical Research Center, Fudan University, Shanghai, China; ^3^Shanghai Key Laboratory of Medical Image Computing and Computer-Assisted Intervention, Shanghai, China

**Keywords:** endoscopic endonasal approach, pituitary adenoma, pseudocapsule, retractor, neurosurgery

## Abstract

**Background:**

The endoscopic endonasal approach and extra-pseudocapsule resection may be the main progress in modern pituitary surgery. However, for pituitary macroadenomas, discerning the pseudocapsule in the posterior plane of the tumor may be difficult. When the anterior-inferior debulking is performed, the early subsidence of the thinning normal pituitary gland and enlarged diaphragm may obstruct the surgical dissection view.

**Method:**

We describe the technique of using a micro retractor for the endoscopic endonasal posterior pseudocapsule resection of pituitary macroadenomas. This micro retractor that was 2 mm in width was placed at the 12 o’clock position on the nostrils, and the end was fixed in the flexible arms of the self-retaining retractor system. The head of the micro retractor elevated the herniated diaphragm sellae in order to continue the posterior pseudocapsule resection of the pituitary macroadenoma.

**Result:**

The technique was performed very easily and no complication was observed.

**Conclusion:**

The use of this micro retractor can increase the view of the posterior margin of the adenomas to facilitate the pseudocapsule dissection.

## Introduction

**Figure 1:**
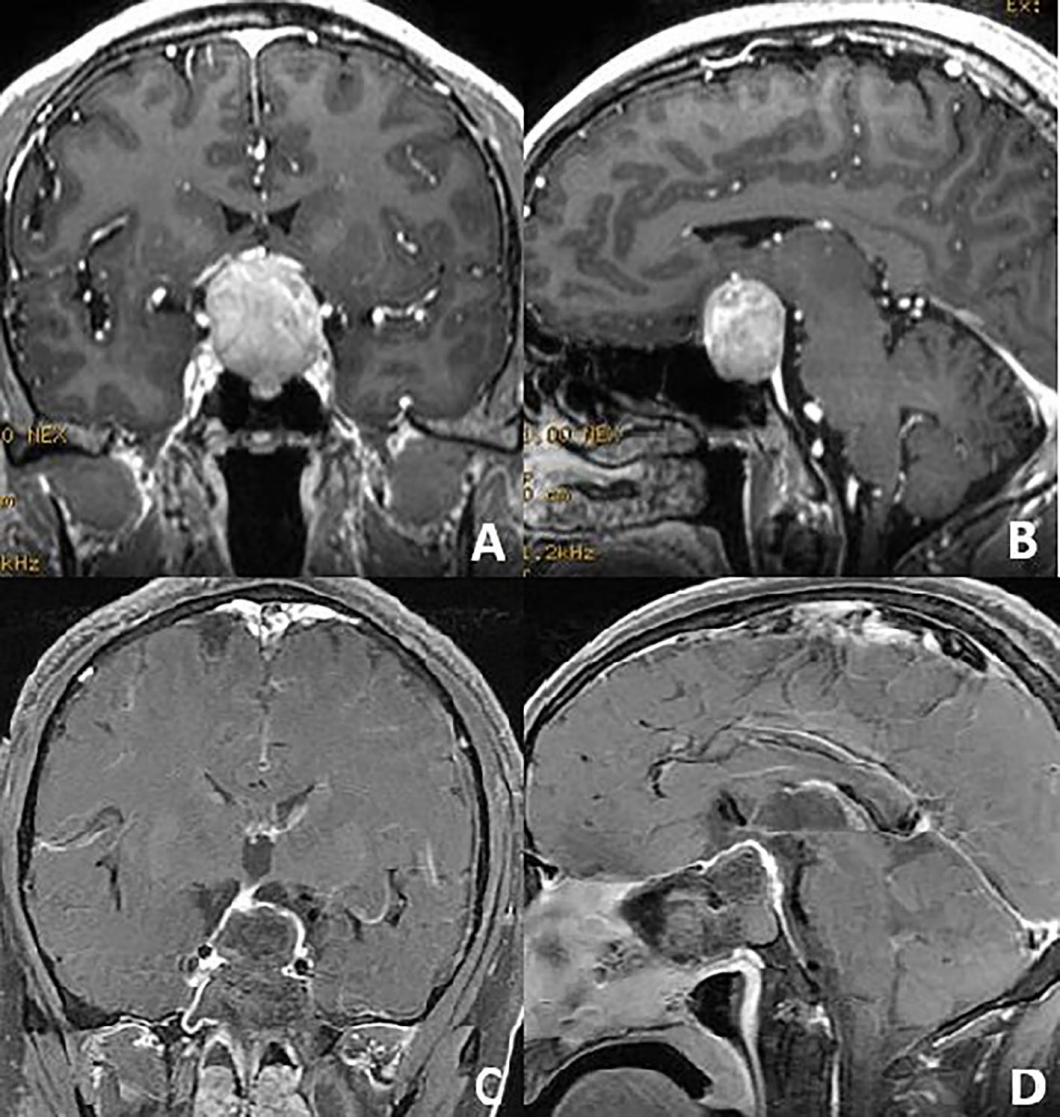
Preoperative coronal and sagittal **(A, B)** contrast-enhanced magnetic resonance T1-weighted images of a non-functional pituitary macroadenoma. After the endoscopic anterior and posterior pseudocapsule dissection, the adenoma was achieved total resection **(C, D)**.

**Figure 2:**
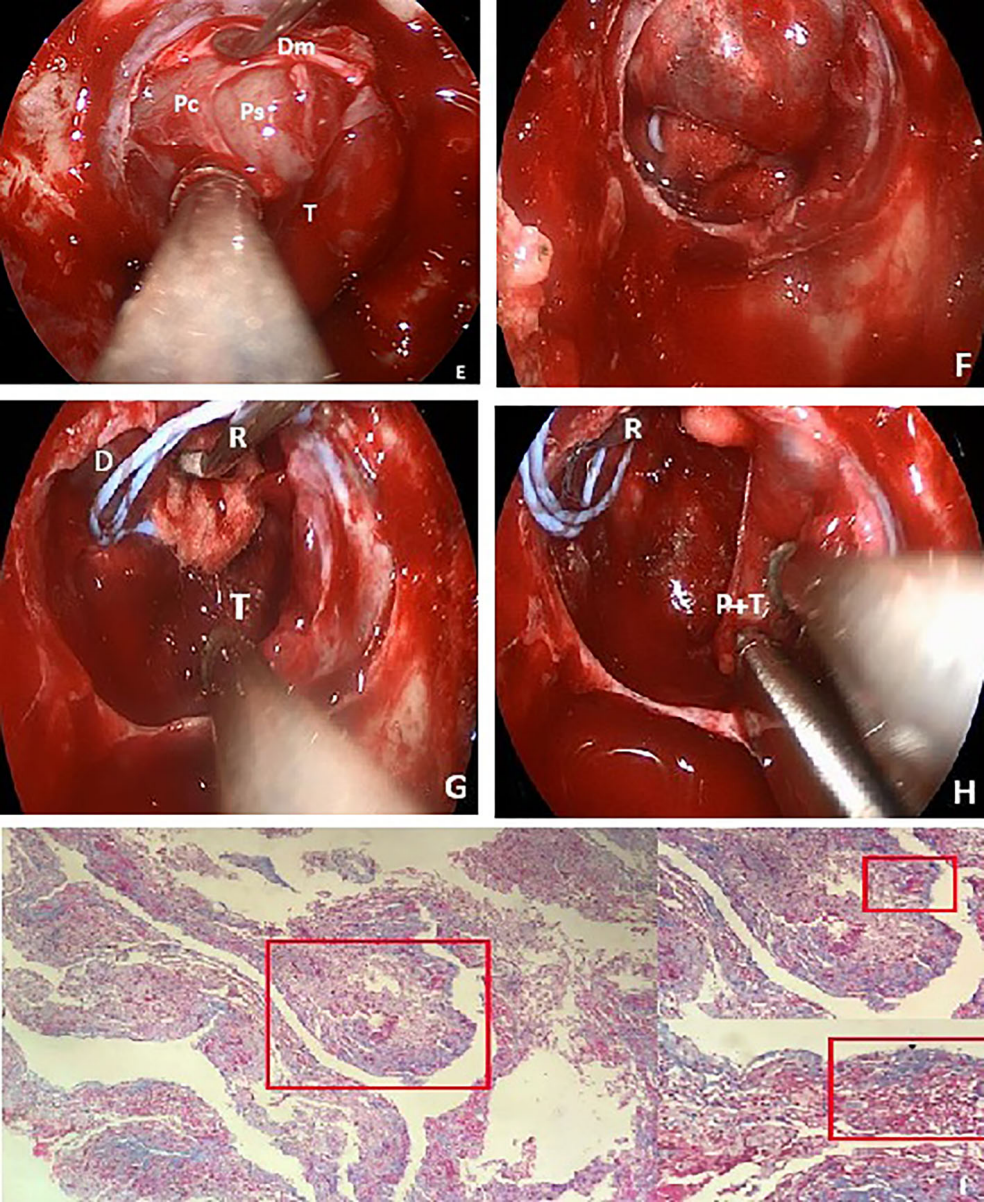
Endoscopic intraoperative views **(E)** showing the anterior layers of the membranes. First is the incised two layers of dura matter (Dm), followed by the pituitary capsule (Pc) and the normal compressed pituitary gland (on the right side). The inner layer is the pseudocapsule (Ps) and the debulking adenoma (T). After the resection, the ballooned diaphragm sellae descended into the sella **(F)**, and it was obstructed to find the posterior fractured pseudocapsule and the remnant adenoma. The micro retractor was used to elevate the descending diaphragm **(G)**. When the vision-blocking diaphragm was pushed up, the posterior fractured pseudocapsule and the remnant adenoma were removed bimanually **(H)**. Pathologic Masson staining **(I)** of this finally removed pseudocapsule and the adenoma. Partial enlarged drawing showed that the pseudocapsule (black arrow) was surrounded around by the adenoma (white arrow).

The advent of endoscopy has begun a new era for pituitary (1). The endoscope and the updated high-definition endoscopes provide a panoramic, close-up surgical view, and allow the eyes of a neurosurgeon to focus not only on the tumor structures but also on the subtle surrounding normal architecture, which has been ignored in the past. The pseudocapsule is one of these structures. The pseudocapsule is not a new concept, as an extraordinarily detailed description was provided by Costello 80 years ago (2). That autopsy research described that many of the adenomas had a thin layer of compressed reticulin, which could easily be distinguished from the pituitary adenoma. Obviously, many neurosurgeons have neglected this small and delicate structure. However, using this pseudocapsule, as the surgical plane for the dissection of an adenoma, is currently gaining increasing attention. This technique improves the gross total resection (3). However, with the increased use of this technique, we found that pseudocapsule dissection might be difficult in the posterior and superior directions for the resection of macroadenomas because of the early subsidence of the thinning normal pituitary gland, and an enlarged diaphragm may obstruct the surgical dissection view after anterior debulking (4, 5). In this report, we describe the use of a micro-retractor to elevate the redundant gland and diaphragm to facilitate the dissection of the posterior pseudocapsule (**Figures 1**, **2**).

### Surgical Technique

**Figure 3:**
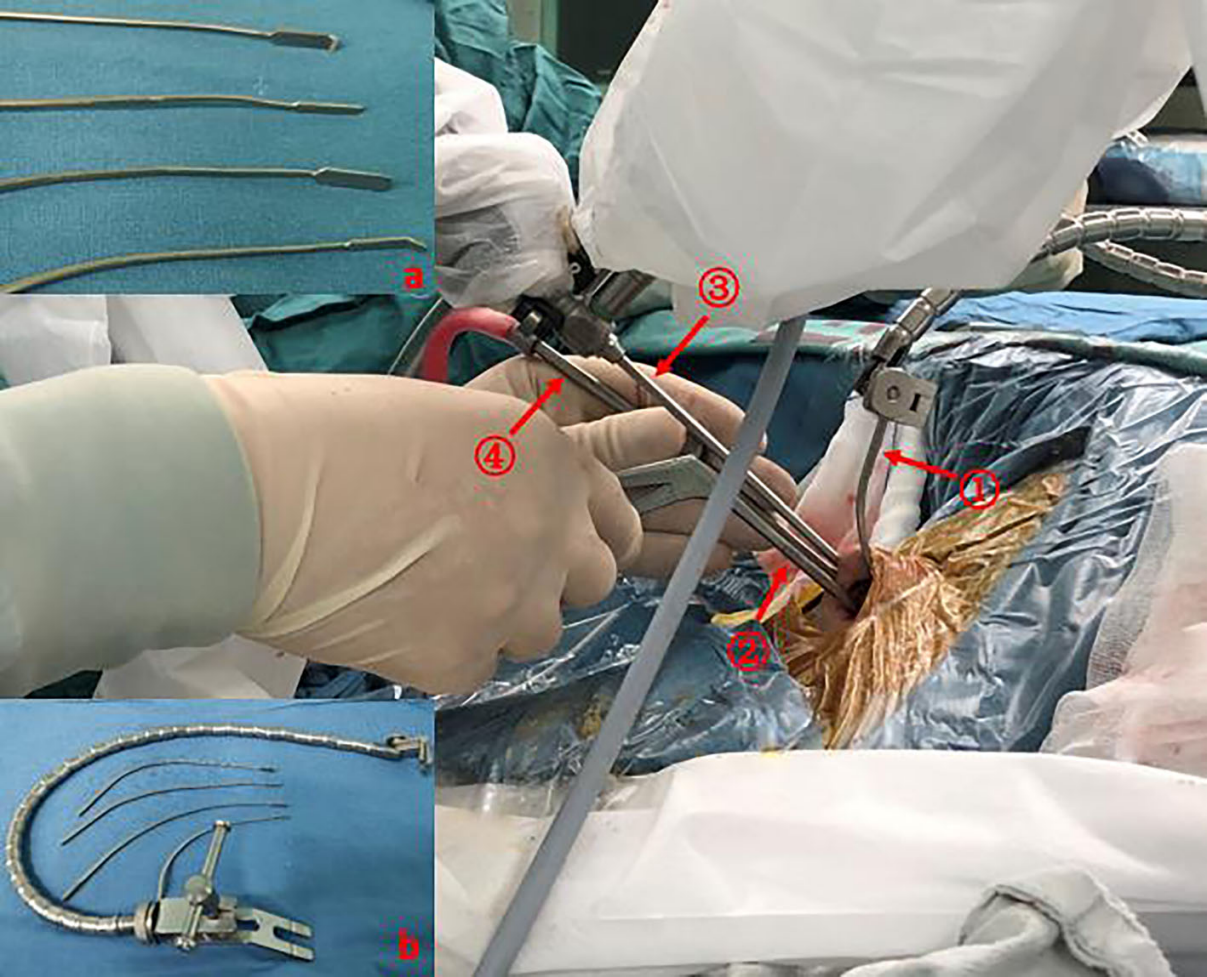
The location of the micro retractor and the other instruments during the endoscopic endonasal approach. The micro retractor (with a self-retaining arm) and the endoscope (with a pneumatic holder) were fixed at the 12 o’clock position on the nostrils. The other instruments could be manipulated bimanually free under the corridor. **(A)** Different lengths and widths of the micro retractor with or without serrated blades. **(B)** The blades were connected with a Leyla flexible self-retaining arm. ①: Micro retractor; ②: Bayonet forceps; ③: Endoscope; ④: Suction.

We used the standard endoscopic endonasal transsphenoidal approach which has been widely described in our and other studies (6). A widely anterior sphenoidotomy was performed, and part of the posterior ethmoid sinus was also removed. Then, the anatomic landmarks around the sella were ascertained. After the entire sella floor was removed, the exposure of the 4-Blue criterion (upper and lower intercavernous sinuses and bilateral cavernous sinus) was required. A No.15 scalpel was used to incise the dura matter (two membranes) with the type, while leaving the underlying pituitary capsule intact. The next incision was made very superficially to incise the pituitary capsule, which is as thin as the wings of a cicada (for macroadenomas, the anterior pituitary gland usually disappears). Blunt microdissection was patiently attempted to develop a surgical plane around the pseudocapsule from the lateral compressed pituitary gland or the medial wall of the cavernous sinus. At this time, a large ring curette was used as a handy tool to peel, rather than scrape, the pseudocapsule. This action informed us that the pseudocapsule dissection was workable. However, in many cases, it was particularly difficult to perform *en bloc* pseudocapsule resection on the macroadenomas. Under these circumstances, suitable debulking was used to create a befitting peripheral margin that was thick enough to provide adequate integrity to the dissection plane. In the real practice, the dissection plane may be ruptured especially in the posterior margin of the pseudocapsule, piecemeal pseudocapsule resection was also typically employed. When the adenoma was gross-totally removed, the thinning normal pituitary gland and enlarged diaphragm would have descended into the surgical view and obstructed the posterior and lateral views. We used a micro retractor (Budde Halo self retaning retractor system, Integra Inc.) which was used to retract the artery during the skull base craniotomy operation to elevate the redundant diaphragm and the gland. The width of the retractor was 2 mm, length was 12 cm, and the end was attached to an automatic retractor (Leyla brain retractor system, Aesculap). This micro retractor was placed at the 12 o’clock position on the nostril to reduce the instrument conflict in the sphenoid sinus (**Figure 3**). When the retractor was in place, a distinct surgical plane between the adenoma and the compressed gland or the redundant diaphragm was visible, and a bimanual microdissection surgical technique was used for a posterior piecemeal pseudocapsule resection could be used for the operator. Multilayer skull base reconstruction was used for the closure.

## Discussion

The use of the histological pseudocapsule to act as a surgical plane to complete the *en bloc* extracapsular resection is the ideal model in pituitary surgery (6). For some pituitary adenomas, this method is feasible. The pseudocapsule consists of several compressed layers of acini and reticulin, which is a type of connective tissue that provides sufficient strength for dissection. As shown in a previous report, the thickness of this membrane can be 0.5–1 mm (7). However, for the majority of adenomas, the *en bloc* resection is difficult. In our opinion, the concept is more important than the surgical skills. The use of the pseudocapsule dissection concept during macroadenoma resection can aid in the performance of pseudocapsule dissection even though the tumor can only be resected in a piecemeal fashion. In terms of macroadenomas, it is easy to perform a pseudocapsule dissection of the anterior and anterior-lateral sides of the tumor when the anatomical layering of the membranes is mastered: the two layers of dura mater, the pituitary capsule, and the pseudocapsule. However, when debulking is performed, further posterior and superior extrapseudocapsular dissection may be difficult because of the early descent of the redundant diaphragm and the compressed gland.

Although the endoscopic approach provides an excellent illumination, high magnification, and a panoramic view of the surgical area, it only provides the possibility of discerning the pseudocapsule; when the redundant diaphragm descends, this possibility disappears. Another “hand” is expected to hold the herniation of the diaphragm. A common approach is to use an aspirator and a small cottonoid to elevate the diaphragm. Additionally, Q-tips (8) or self-retaining retractors (5) have also been reported to solve the herniation problem. However, these techniques are still associated with the problems of a non-bimanual dissection or a limited working space.

The abovementioned micro retractor blade system has been used to move cerebral blood vessels or delicate neurological structures during cranial procedures (9). The micro retractor is part of the Budde Halo self-retaining retractor system (Integra Inc., USA). The optimum use for this retractor is to avail the surgeon of both hands for a bimanual procedure in the supraclinoid or cerebellopontine angle regions. In our opinion, this self-retaining retractor can also free the hands of the surgeon during the endoscopic endonasal approach. The flexible articulate arm can be placed in any direction to facilitate the procedures in the narrow sella region. Micro- and vimineous blades can hold the diaphragm to facilitate a bimanual posterior pseudocapsule margin dissection. When the retractor blade is fixed in the 12 o’clock position on the nostril, the lower working space and surgical freedom are never affected. This retractor blade can be recombined with the Leyla brain retractor, which can be attached to the operating bed. This recombination reduces the obstruction of the arch of the Budde Halo system and avoids the connection of the head clamp (10).

The reason why the posterior pseudocapsule surgical plane is hard to expose is partly because of the obstruction of the macrovolume of the adenoma and is partly due to the early descent of the redundant diaphragm and the compressed gland. The debulking procedure and the micro retractor system can solve this difficulty. After anterior and lateral pseudocapsule dissection, even when the pseudocapsule is fractured, the remnant posterior and superior planes of the pseudocapsule can be clearly exposed to avoid blind curettage and remnant tumors.

## Conclusion

This micro retractor can increase the view of the posterior margin of the adenomas to facilitate the pseudocapsule dissection. Increasing the clinical use of this micro retractor is expected during the endoscopic endonasal surgery when a “third hand” is needed.

## Data Availability Statement

The original contributions presented in the study are included in the article/supplementary material. Further inquiries can be directed to the corresponding author.

## Author Contributions

All authors contributed to the article and approved the submitted version.

## Funding

Foundation of Science and Technology Commission of Shanghai Municipality (21ZR1413100). 

## Conflict of Interest

The authors declare that the research was conducted in the absence of any commercial or financial relationships that could be construed as a potential conflict of interest.

The handling editor declared a shared affiliation with the authors at time of review.

## Publisher’s Note

All claims expressed in this article are solely those of the authors and do not necessarily represent those of their affiliated organizations, or those of the publisher, the editors and the reviewers. Any product that may be evaluated in this article, or claim that may be made by its manufacturer, is not guaranteed or endorsed by the publisher.
